# Effect of Pre-Shear on Agglomeration and Rheological Parameters of Cement Paste

**DOI:** 10.3390/ma13092173

**Published:** 2020-05-08

**Authors:** Mareike Thiedeitz, Inka Dressler, Thomas Kränkel, Christoph Gehlen, Dirk Lowke

**Affiliations:** 1 Centre for building materials, Technical University of Munich, 81245 München, Germany; thomas.kraenkel@tum.de (T.K.); gehlen@tum.de (C.G.); 2 Institute of Building Materials, Concrete Construction and Fire Safety, Technische Universität Braunschweig, 38106 Braunschweig, Germany; d.lowke@ibmb.tu-bs.de

**Keywords:** rheology, pre-shear, flocculation, FBRM, state of flocculation

## Abstract

Cementitious pastes are multiphase suspensions that are rheologically characterized by viscosity and yield stress. They tend to flocculate during rest due to attractive interparticle forces, and desagglomerate when shear is induced. The shear history, e.g., mixing energy and time, determines the apparent state of flocculation and accordingly the particle size distribution of the cement in the suspension, which itself affects suspension’s plastic viscosity and yield stress. Thus, it is crucial to understand the effect of the mixing procedure of cementitious suspensions before starting rheological measurements. However, the measurement of the in-situ particle agglomeration status is difficult, due to rapidly changing particle network structuration. The focused beam reflectance measurement (FBRM) technique offers an opportunity for the in-situ investigation of the chord length distribution. This enables to detect the state of flocculation of the particles during shear. Cementitious pastes differing in their solid fraction and superplasticizer content were analyzed after various pre-shear histories, i.e., mixing times. Yield stress and viscosity were measured in a parallel-plate-rheometer and related to in-situ measurements of the chord length distribution with the FBRM-probe to characterize the agglomeration status. With increasing mixing time agglomerates were increasingly broken up in dependence of pre-shear: After 300 s of pre-shear the agglomerate sizes decreased by 10 µm to 15 µm compared to a 30 s pre-shear. At the same time dynamic yield stress and viscosity decreased up to 30% until a state of equilibrium was almost reached. The investigations show a correlation between mean chord length and the corresponding rheological parameters affected by the duration of pre-shear.

## 1. Introduction

The rheology of cementitious pastes determines the flowability of concrete. Especially in modern concretes like Ultra High Performance Concrete (UHPC) and Self-Compacting Concrete (SCC) with a high amount of fine particles (d_max_ < 125 µm), the knowledge of rheological parameters such as yield stress, viscosity and structural build-up is essential for a proper placement process. Quantitative fundamental knowledge about the characteristics on a microscale helps understanding and estimating the qualitative rheological performance, namely mixing, pumping and placing [1,2,3,4,5,6]. Nevertheless, fundamental microscopic insights on an absolute scale are difficult to achieve: many parameters like the granulometry of particles, i.e., the particle size distribution and particle shape, solid fraction and chemical composition of the cement as well as supplementary cementitious materials or fillers have an effect on the rheological parameters [7,8].

In this study the correlation between the state of agglomeration and rheological parameters are investigated. One main influencing factor on rheological behavior is shear, varying in duration and intensity and thus leading to (micro-) structural changes and modified flow characteristics. This effect is particularly important in the context of rheological measurements on cementitious suspensions, since every variation of the shear history before the start of a measurement causes a change in the rheological parameters. Consequently, the effect of pre-shear time on agglomeration kinetics and rheological parameters is investigated. Furthermore, these findings could be a base for further fundamental research concerning the effect of shear history (e.g., during mixing, pumping, spraying) on the agglomeration status that itself affects the rheological behavior of cementitious suspensions during casting (e.g., workability, form filling ability).

## 2. From Microscopic to Macroscopic Scale: Agglomeration and Rheology

### 2.1. Effect of Particle Structuration on Rheology

Cement paste is a two-phase colloidal suspension consisting of particles as disperse phase and a carrier liquid, containing particles ranging from nanometers to several hundreds of micrometers in size. Due to high particle concentrations above the percolation threshold, cement paste is a non-Newtonian suspension with a yield stress and a shear-rate dependent viscosity.

The presence and magnitude of particle networks is based on interparticle forces. Those determine the rheological parameters like yield stress and viscosity as well as the reversible network breakage and build up, called thixotropy [9,10,11]. Colloidal interactions and chemical nucleation hydration effects of the cement particles lead to particle agglomeration. The strength of interparticle networks due to agglomeration and coagulation is dependent on interaction forces. Possible interaction forces are hydrodynamic forces, Brownian motion of particles smaller than 10 μm due to thermal randomizing activity and colloidal interaction forces. These forces are either attractive or repulsive. Attractive forces are Van-der-Waals-forces and electrostatic attraction, whereas repulsive forces are caused by electrostatic repulsion and steric hindrances [2,4,12,13]. Depending on the sum of collision and non-contact colloidal forces, particle interactions either lead to stable dispersions in the fluid by virtue of predominant repulsion forces or the formation of agglomeration networks [1,14]. Generally, cementitious suspensions without superplasticizer are the latter. 

Shear-induced microstructural changes of the particle network can either lead to shear-thinning or shear-thickening behavior, mainly depending on the solid concentration of the suspension. Colloidal suspensions tend to shear-thinning behavior caused by the shear-induced breakage of agglomerates in the particle network, whereas densely packed suspensions tend to shear-thickening behavior due to increasing frictional forces [2,15]. With an adjusted granulometry and herewith packing density, the viscosity can be decreased tremendously while maintaining the solid volume fraction [16]. Still, with decreasing particle diameter while remaining at the same solid volume fraction, the sum of interparticle forces tends to increase due to a percental increase in the specific surface area [13,17,18]. Since viscosity indicates the resistance against shear, it is a shear-rate dependent apparent viscosity. On the contrary, viscosity calculations are often either particle structure based or shear dependent equations. Particle structure based equations take the relative solid volume fraction $\phi_{rel}$ (ratio of the solid concentration to the maximum solid concentration $\Phi /\Phi_{max}$) into account [1,15,19] or calculate the apparent particle size distribution as well as particle interactions in dependence of the solid volume fraction [20,21]. These equations are mainly valuable for ideally sphered particle suspensions. Shear-rate dependent viscosity equations state a zero-shear viscosity, following a percental decrease in viscosity with increasing shear. Although much research has focused on the apparent shear-rate dependent viscosity of suspensions, it is mainly valuable for monodisperse, ideally sphered or inert particle structures [2,14,15,18,19,21,22]. Particularly, further research effort is needed for the analysis of microstructure, mechanics of deformation and orientation, particle friction and agglomeration processes, especially during shear for non-spherical and reacting particle suspensions like cement paste.

### 2.2. Effect of Pre-Shear Time on Rheological Parameters

In rheometric investigations, the apparent particle structure affects the measured rheological parameters. Due to agglomeration processes, aggregate growth leads to a change in the apparent particle size distribution. The degree of agglomeration therefore determines the effective rheological parameters. With changing shear history of agglomerated suspensions, the rheological parameters are not directly comparable. The applied shear in a rheometer is generally not sufficient to break all already built agglomerates of a particle network. Therefore, the paste has to be presheared with an external mixer. With increasing shear intensity or pre-shear-time, the mean agglomerate size decreases [23,24], while partly shear-induced particle collisions might produce reformation of networks [11]. If the foremost particle network is completely de-flocculated or the introduced shear is limited and not strong enough to break further agglomerates, equilibrium is obtained [11,23,25,26]. After shear (at rest), particles will agglomerate again. Thus, the particle network is a function of the cement paste’s shear history (i.e., mixing) [18,20,27]. In references [26,28] the correlation between rheological parameters and the effective mean diameter of agglomerates in dependence of shear history was investigated over time. Still, research focusing on the effect of the shear history on particle network structuration and incorporated rheological parameters, has not been conducted sufficiently on an absolute scale. To ensure the reproducibility and comparability of rheometric results, a proper reference state of agglomeration and thus the apparent particle network has to be found in order to enable correct interpretation of rheometric results and as input parameter for further approaches for modeling rheological parameters [29,30].

### 2.3. Methods to Investigate Agglomerate Size Distributions of Cement Paste

There is a variety of methods measuring agglomerate size distribution. Yang et al. [31] performed sedimentation studies to qualitatively observe the agglomerate size of cement suspensions. According to Stokes law particles in a dilute suspension settle freely at an equilibrium speed proportional to the square of the radii of the particles. Larger particles settle faster, while smaller particles remain suspended longer. Particles in flocculated or coagulated suspensions consist of settling agglomerates rather than individual particles. If these agglomerates are large, they are considered as large particles and settle rapidly in the suspension. However, a short-term change of the state of agglomeration is not detectable with this method due to the long duration of the experiments. In addition, investigations on fluids with very high solid volume fractions, which are usually prevalent in cement paste, cannot be carried out with this method.

The cryo-preparation of suspensions and their subsequent investigations in the scanning electron microscope [32] or by means of focused ion beam nanotomography [33] allows statements on the structure and agglomeration state at different times. However, this procedure is time-consuming. In addition, these methods require firmly fixed particles. This is usually conducted by a fast-freezing process (e.g., in liquid nitrogen). This process raises the possibility of alterations in particle arrangements induced by ice crystals produced during the freezing process or during any subsequent manipulations used to remove the frozen water. Since larger samples do not freeze fast enough in the core area, the desired microstructure state can no longer be investigated. It is a decisive limitation of these methods that they cannot be carried out during shearing the suspension; hence, short-term time- and shear rate-dependent changes in the agglomerate size distribution cannot be detected. However, short-term structural changes are of crucial importance for the flow behavior of suspensions with high solid volume fractions, especially of cementitious suspensions [4]. The method of electroacoustics [34] enables the in situ measurement of particle size distributions, whereby a limited suitability at very high solid fractions is given. In addition, not the diameters of the agglomerates, but those of the primary particles are measured. Due to the named limitations, the mentioned methods for the determination of the agglomerate size distribution are only conditionally suitable for the investigation of practical relevant cement pastes under shear. Further experimental methods are mentioned, e.g., in reference [35].

Optical laser-assisted methods are suitable for the rapid in situ detection of an agglomerate size distribution even in sheared and highly packed systems. To measure the chord length of the agglomerate, the focus of the laser rotates during the measurement, e.g., FBRM (Focused Beam Reflectance Measurement) or 3D ORM (Three Dimensional Optical Reflectance Measurement). Measurements were for example carried out on sheared cement pastes [36,37,38,39], limestone powder suspensions [40] or on crystallization kinetics, determined in solutions relevant to the pharmaceutical industry [41].

## 3. Materials and Methods 

### 3.1. Concept and Procedure of Investigation

The investigations pursued the main goal to link the state of agglomeration and rheological parameters. Therefore, the effect of pre-shear time on agglomeration kinetics and rheological parameters of cement paste are investigated. The agglomerate size during shear were measured using a FBRM-probe. Rheological parameters were obtained afterwards using a rheometer.

Various pre-shear times (30 s, 90 s and 300 s) were applied on cement pastes with practical relevant solid volume fractions (*ϕ* = 0.45, 0.48 and 0.52). The pastes contained a PCE-based superplasticizer with an adjusted amount for a mini-slump flow 4.5 min after water addition of 250 ± 5 mm using the Hägermann cone (d_1_ = 70 mm, d_2_ = 100 mm, h = 60 mm, according to DIN EN 12350-8 [42]). The pastes are named according to their solid volume fraction and if applicable, the pre-shear time added in seconds. Moreover, one reference cement paste without superplasticizer and a solid volume fraction of 0.45 was used (0.45_xSP).

**Figure 1:**
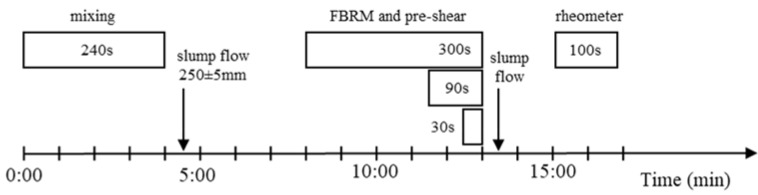
Time sequence of the investigations.

The time sequence of the investigations is shown in Figure 1. The mini-slump flow was measured immediately after mixing, which was 4:30 min after water addition. After the mixing procedure, cement paste rested and was subsequently sheared at the cement paste age of t = 8, 11.5 or 12.5 min after water addition, respectively. Simultaneously, the FBRM-measurements were performed. The pre-shear and FBRM-measurements were stopped at 13 min. Next, 13.5 min after water addition, another mini-slump flow was conducted. Then, at t = 15 min after water addition, the rheometer experiments started.

### 3.2. Mixture Preparation and Composition

Cement paste containing Ordinary Portland Cement (CEM I 42.5 R) and demineralized water were prepared according to DIN EN 196-1 [43]. The cement was stored at 20 °C without solar irradiation. The used mixer (ToniMix Basic, Toni Technik, Berlin, Germany) is standardized for the production of cement paste and mortar acc. to DIN EN 196-1 [43]. For each mixture, a testing volume of 1.0 l was prepared. Water was added to cement immediately before starting the mixing process. After slow mixing for 90 s at 140 ± 5 rpm, superplasticizer was added during 30 s of rest. Then, the paste was mixed for another 90 s at 285 ± 10 rpm. The mixture proportions of the pastes are shown in Table 1.

### 3.3. FBRM-Measurements

FBRM is an in-situ technique for characterizing the particle size distribution of highly packed suspensions [44]. It consists of a rotating laser beam, leading to a circular moving focus point in close vicinity to the probe’s window. If a particle is hit by this focus point, light is scattered back and can be registered by a detector. The tangential velocity of the beam and its speed are known and are expected to distinguish significantly from the particle’s velocity within the cup. Therefore, the width of the particle or the agglomerate can directly be calculated from scattering time and rotation speed. This calculated value equals the chord length of the particle or agglomerate. The chord length distribution is directly linked to the size distribution of agglomerates [45]. In the following, the median agglomerate chord length *d*_50_ (named in the following: agglomerate size) is used to characterize the state of agglomeration.

**Figure 2:**
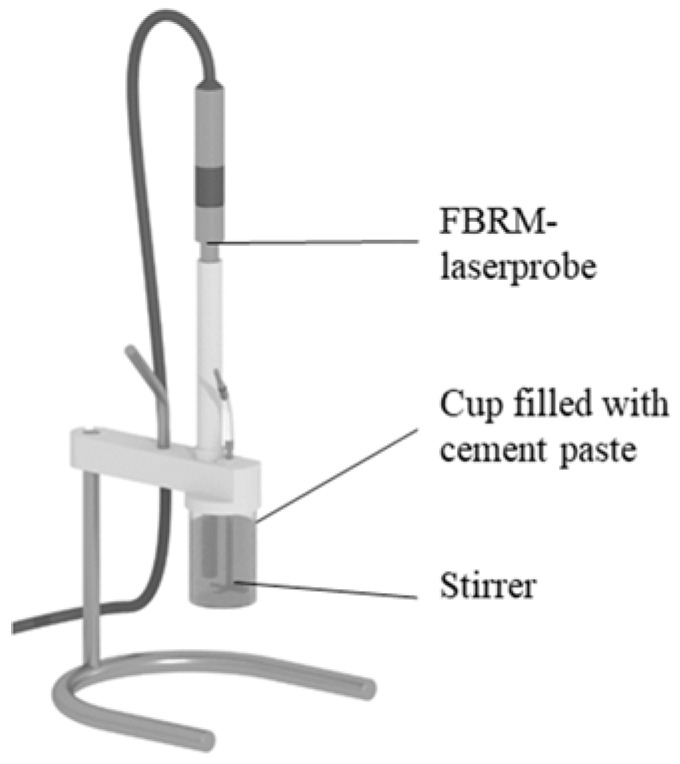
Experimental setup of the FBRM-measurements.

After the mixing procedure, 0.4 L of the paste was filled into a separate cup. In this cup, material is sheared with a four bladed stirrer, compare setup in Figure 2. When starting to shear the cement paste with 1700 rpm, FBRM-measurements (G400, Mettler Toledo, Gießen, Germany) were started to obtain the agglomerate size distribution with a measurement frequency of f = 0.5 Hz. It is worth noting that applied shear acting on cement paste is not only depending on pre-shear time but also on other factors, such as the shape of the stirrer and geometry of the cup. However, these factors were kept constant and are therefore not taken into account. To determine the state of agglomeration in the beginning and in the end of shear, the first and the last 5 measurement values were arithmetically averaged.

### 3.4. Rheological Measurements

**Figure 3:**
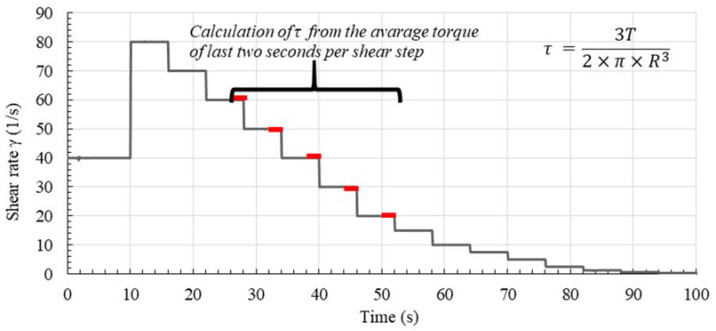
Shear protocol for rheometric measurements.

For the rheological measurements an MCR 502 rheometer (Anton Paar, Graz, Austria) with serrated plates (diameter = 50 mm) was used. The serrated plates prevent from wall slip. The gap between the plates was set to 1 mm for homogenized sample conditions over the whole gap and the possibility of a fully sheared gap. In the beginning, 10 s of shear at 40 s^−1^ were applied to break agglomerates and thus to enable a defined state of agglomeration in the sample at the beginning of the rheometric measurements. Then, a shear profile with decreasing steps of shear was applied (Figure 3): In 15 steps, lasting 6 s, respectively, the torque was measured with decreasing shear rates from 80 s^−1^ to 0.02 s^−1^. Each step contained 60 data points, i.e., one data point each 0.1 sec. The average torque was calculated from the torque values of the last two seconds for each shear step, which could be assumed as equilibrium state. The shear stress was calculated following the equation given in Figure 3, compare [46]. The dynamic yield stress *τ*_0,*d*_ for each flow curve was calculated following the Bingham regression for the steady state between applied shear rates $\dot{\gamma }$ of 20 s^−1^ and 60 s^−1^. The viscosity $\mu_{P}$ was taken as plastic viscosity for the same flow region.

## 4. Results and Discussion

In the following Section 4.1 and Section 4.2, the effect of pre-shear on the state of agglomeration and on rheological parameters is presented. Then, the results of both sections are correlated in Section 4.3. In Table 2 the obtained results are summarized. These comprise the measured median chord lengths *d*_50_ after shear measured with FBRM, the mini-slump flow values SF (mm) at 13.5 min after water addition as well as the calculated yield stresses *τ*_0,*B*_ (Pa) and plastic viscosities $\mu_{P}$ (Pa.s) from rheometer data for all test series. Both the yield stress *τ*_0,*B*_ and the plastic viscosity values μ were calculated as Bingham regression for the shear region from $\dot{\gamma }$ = 20–60 s^−1^.

### 4.1. Effect of Pre-Shear on State of Agglomeration 

**Figure 4:**
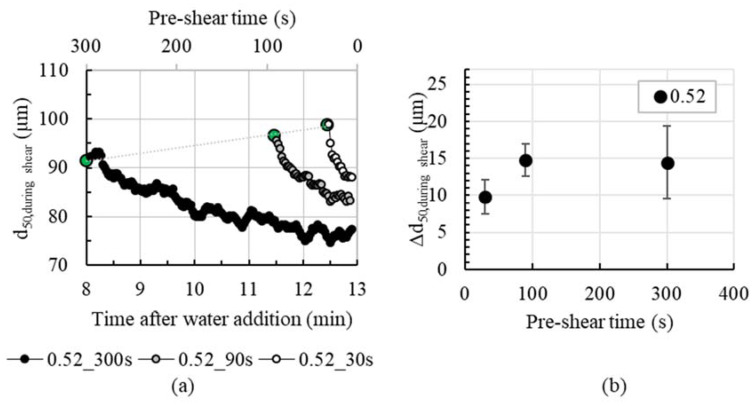
(**a**) Agglomerate size d_50_ decreases during shear for pre-shear times of 30 s, 90 s and 300 s (shown here with a moving average with 5 values); (**b**) summarized agglomerate size decrease *Δd*_50_ from beginning to end of shear for various pre-shear times (n = 2); both examples are shown for cement paste 0.52.

Figure 4a shows the agglomerate size over pre-shear-time and time after water addition, respectively, with the example of cement pastes with a solid fraction of 0.52. The initial agglomerate size of the cement pastes before shear increases with decreasing intended pre-shear time, i.e., increasing age of the cement paste. The agglomerate size before applying 30 s of shear (12.5 min after water addition) amounts to approximately 100 µm. The smallest mean agglomerate size is approximately 91 µm for cement paste 0.52_300s (measured 8 min after water addition, compare larger circle symbols in Figure 4a). For an increase in agglomerate size with increasing cement paste age, there are two reasons:

- (a) Time at rest: Shorter time after mixing (deflocculated state) means less time to form agglomerates;
- (b) Hydration: The initial phase of hydration is dominated by the reaction of C_3_A with sulphate to ettringite. The ettringite develops around the unhydrated sections of the cement and forms small particles—approximately sized in the range of 100–500 nm [33]—meaning that the particle itself becomes marginally larger over time and so the agglomerates increase.

It is assumed that the effect (a) dominates the effect (b), since the growing ettringite are three magnitudes lower than the observed grain growth.

When cement paste is stirred, the agglomerate size continuously decreases, Figure 4a. The higher the pre-shear time, the higher the decrease in mean agglomerate size, i.e., the difference between agglomerate size before and after shear *Δd*_50_, see Figure 4b. The agglomerate size decreases by about 10 µm for 30 s of shear and nearly 15 µm for 300 s of shear.

**Figure 5:**
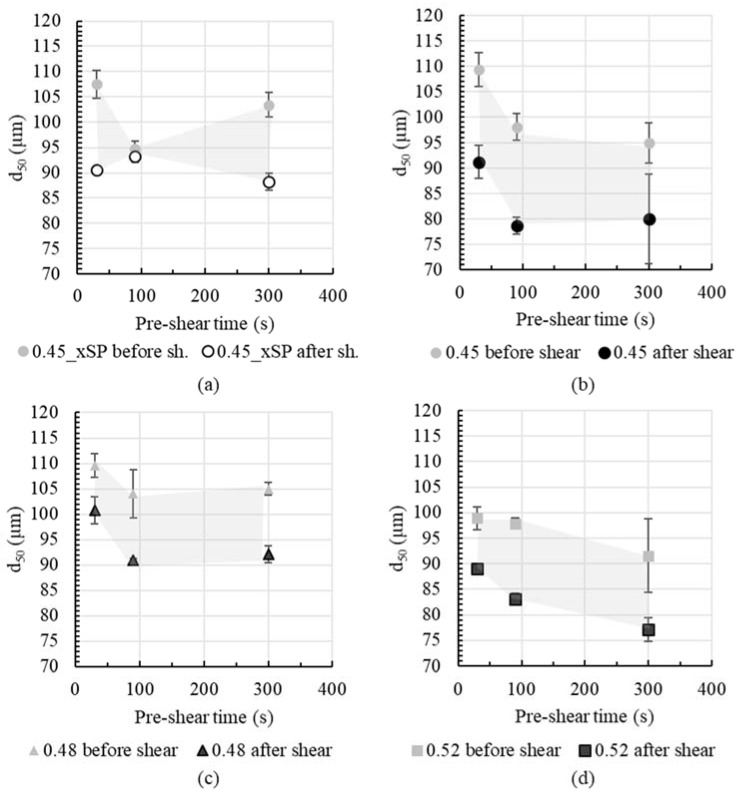
Agglomerate size *d*_50_ of cement paste with various solid volume fractions (**a**) 0.45_xSP (without superplasticizer), (**b**) 0.45, (**c**) 0.48 and (**d**) 0.52 over pre-shear time before and after applied shear. The grey area indicates the agglomerate size decrease during shear.

Figure 5 shows the summary of agglomerate size over pre-shear time before and after shear for all cement pastes. When comparing cement paste with (0.45) and without superplasticizer (0.45_xSP) it is observed that cement paste with superplasticizer reveals a higher agglomerate size decrease (from beginning to end of shear), expressed in a larger distance of the symbols before and after shear, compare Figure 5a,b. For all cement pastes, an increase in pre-shear time from 30 s to 300 s comes along with a decrease in agglomerate size after shear, compare dark circled symbols in Figure 5a–d. This is particularly pronounced for cement pastes with superplasticizer. It is assumed that the usage of superplasticizer results in a reduction in attractive interparticle forces. Hence, only weak agglomerates are formed, which can be broken more easily under shear compared to cement pastes without superplasticizer.

**Figure 6:**
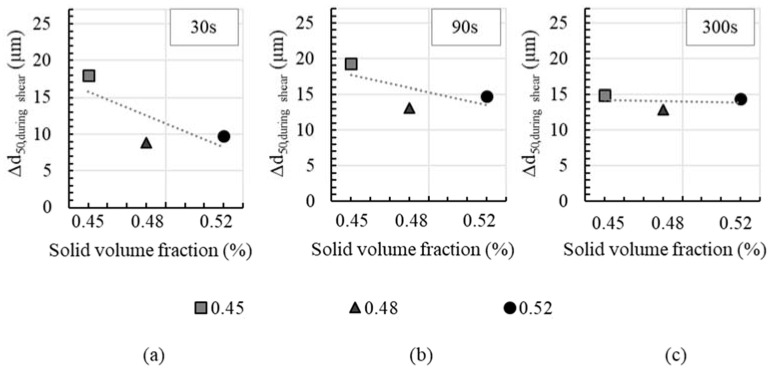
Agglomerate size decrease *Δd*_50_ due to shear over solid volume fraction; shown for pre-shear times of (**a**) 30 s; (**b**) 90 s and (**c**) 300 s. Indicated trend for cement paste with superplasticizer only.

Moreover, it is observed that the solid volume fraction has an effect on agglomerate size decrease *Δd*_50_. In Figure 6 the agglomerate size decrease *Δd*_50_ (reduction in agglomerate size from beginning to end of shear) is shown for all cement pastes with superplasticizer for a pre-shear time of 30, 90 and 300 s. At high solid volume fractions, *Δd*_50_ tends to decrease. This effect is particularly distinct for short pre-shear times, as shown in Figure 6a for 30 s of pre-shear. It is assumed that the particle network within a cement paste with high solid volume fraction, i.e., with a high number of particle contacts, can only be broken up to a lesser extent during short pre-shear periods than is the case at lower solid concentrations and correspondingly lower numbers of particle contacts. At a pre-shear time of 90 s, the shear energy input is high enough to break up more agglomerates even in pastes with high solid volume fractions. This leads to an increase in *Δd*_50_ at high solid volume fractions and thus to a lower dependence of Δd_50_ on the solid volume fraction, Figure 6b. With high pre-shear times of 300 s, the effect of solid volume fraction on *Δd*_50_ is not pronounced, Figure 6c. The agglomerates are broken up almost in the same degree for all solid volume fractions. In addition, it can be recognized that *Δd*_50_ is lower at low solid volume fractions than with shorter pre-shear times. This is mainly attributed to the fact that with increasing pre-shear time the age of the cement paste and thus the duration of the structural build-up at rest decreases, compare Figure 1. Due to the shorter time at rest, the agglomerate size at the beginning of shear is significantly lower. This means that, starting from a smaller agglomerate size, the degree of agglomerate break-up is reduced.

### 4.2. Effect of Pre-Shear on Rheological Parameters

**Figure 7:**
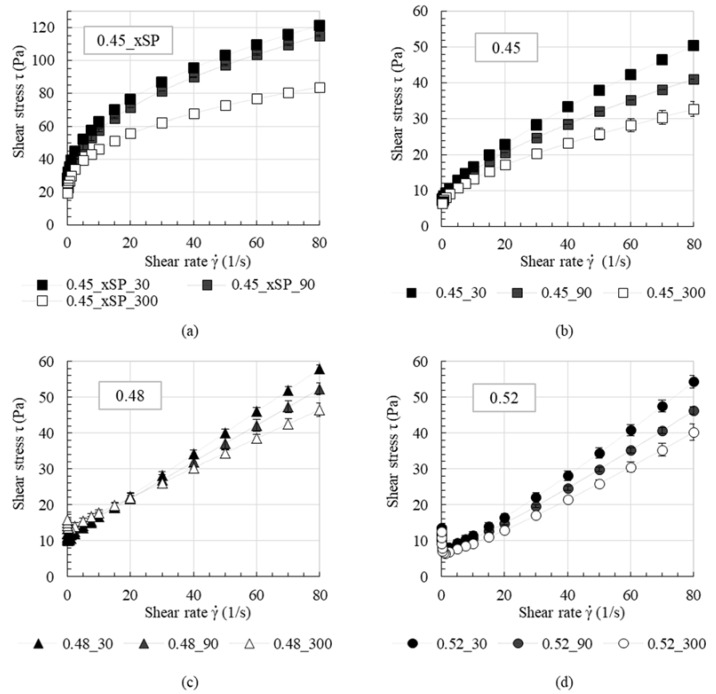
Flow curves of series 0.45_xSP (**a**), 0.45 (**b**), 0.48 (**c**) and 0.52 (**d**) as a function of pre-shear time.

The average flow curve of three repetitions and its standard deviation for each cement paste are shown in Figure 7 for all test series. The standard deviation was calculated for a small sample number using the Gaussian distribution. Fundamentally, it can be observed that with increasing pre-shear time, the resulting shear stress during the measurement decreases. For the test series without PCE (0.45_xSP), a strong decrease in shear stress especially occurs for pre-shear times of 300 s, where the resulting shear stress at high shear rates decreases by about 30%, compared to 30 s of pre-shear time. This does not apply for the other test series. Series 0.45 shows its strongest decrease in shear stress until 90 s with around 23%, compared to the reference shear stress with 30 s of pre-shear. Interestingly, the pastes with a high solid volume fraction of 0.48 and 0.52 show less pronounced changes in shear stress. It is also worth noting that the rheological behavior changes from shear thinning for a solid volume fraction of 0.45 to slightly shear thickening for a solid volume fraction of 0.52. For a solid volume fraction of 0.48, nearly Bingham-like rheological behavior is measured.

**Figure 8:**
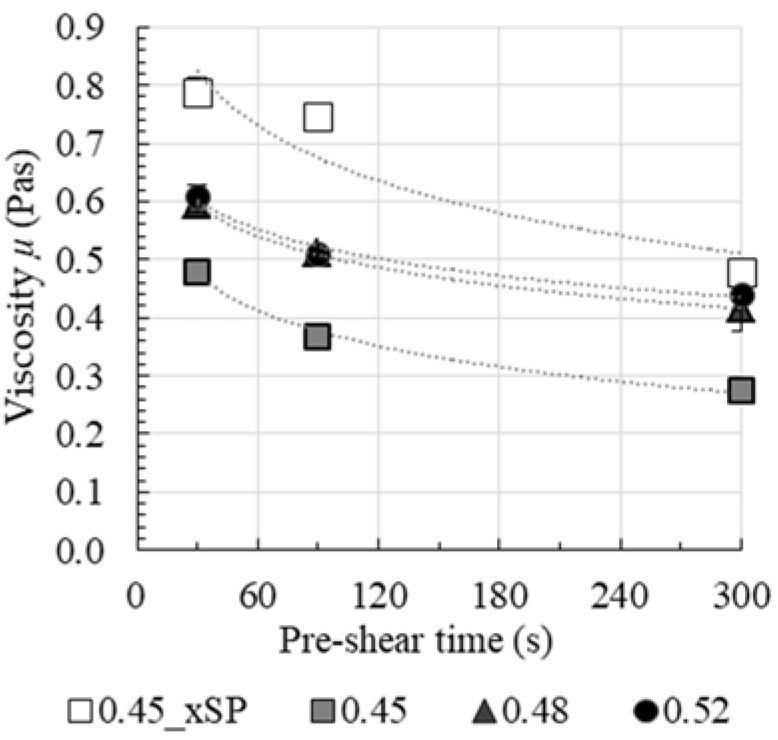
Effect of pre-shear time on viscosity.

**Figure 9:**
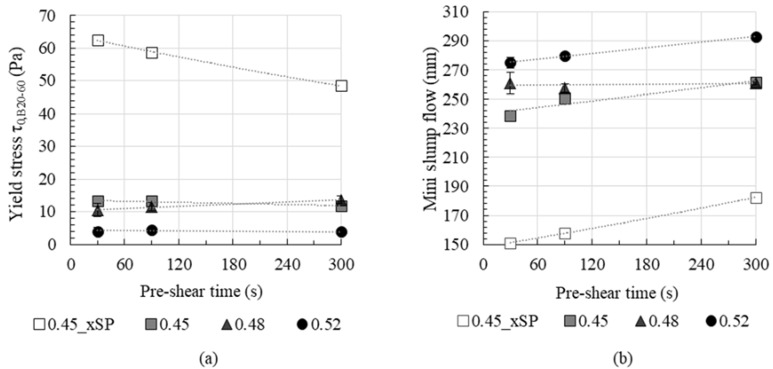
Effect of pre-shear time on yield stress (**a**) and mini-slump flow after shear (**b**).

The rheological parameters dynamic yield stress τ_0,B_ and plastic viscosity $\mu_{P}$ were calculated as Bingham regression in a shear region of $\dot{\gamma }$ = 20–60 s^−1^ for the resulting flow curves. The dependence of rheological parameters on the pre-shear time is shown in Figure 8 and Figure 9.

In Figure 8, the effect of pre-shear time on viscosity is shown for all test series. It is apparent that the plastic viscosity $\mu_{P}$ decreases with increasing pre-shear time. The decrease in $\mu_{P}$ with increasing pre-shear time differs for pastes with and without superplasticizer. Without superplasticizer (0.45_xSP) the effect of agglomerate breakage leads to a decrease in $\mu_{P}$ of nearly 40% after 300 s of pre-shear time compared to the viscosity after 30 s. By comparing cement pastes with superplasticizer, it becomes apparent that the percentage decrease in the more densely packed suspensions 0.48 and 0.52 is less pronounced, but also leads to values of about 30% of decrease after 300 s. An equilibrium viscosity with regard to pre-shear is not reached. Therefore, it is assumed that induced shear is not sufficient for a comprehensive breakage of the agglomerates in the cementitious suspension.

Furthermore, the results clearly show that viscosity is affected by the solid volume fraction *ϕ_act_* as well as the use of superplasticizer. Without superplasticizer, a strong cement particle network with a high degree of agglomeration leads to a high viscosity compared to the paste with identical solid volume fraction with superplasticizer. This is reasonable due to the dispersing effect of the superplasticizer. Despite the same theoretical yield stress (through an adjustment of the mini-slump flow), the viscosity of the paste series 0.45, 0.48 and 0.52 varied due to different solid volume fractions. With an increasing solid volume fraction, viscosity also increases.

The effect of pre-shear time on Bingham yield stress calculated for shear rates between 20–60 s^−1^ is shown in Figure 9a. Additionally, the mini-slump flow measurements are shown in Figure 9b. According to reference [47] the yield stress can be calculated from mini-slump flow. Increasing slump flow comes along with a decrease in yield stress. Thus, for an estimation of the effect of pre-shear on the resulting yield stress, the change in the mini-slump flow values is a good indication.

For the cement paste without superplasticizer, the dynamic yield stress *τ*_0,*B*_ decreases significantly with increasing pre-shear time, as shown in Figure 9a. However, for the cement pastes with superplasticizer no significant effect of prehearing time on dynamic yield stress could be observed. In contrast, mini-slump flow values increase with pre-shear time for pastes with and without superplasticizer. It is assumed that shear load during measurement of yield stress or slump flow plays an important role. During the measurement in the rheometer, high shear rates up to 80 s^−1^ are applied to the cement paste sample, resulting in an effective dispersion of the weak agglomerates in the superplasticized cement pastes. This deagglomeration during the rheometer measurement seems to dominate the effect of pre-shear. On the contrary, during the mini-slump flow measurement only low shear rates are prevalent in the cement paste sample, which does not result in a significant change of the agglomerate structure during the measurement. Thus, the effect of pre-shear is more pronounced in slump flow measurements than in rheometric investigations. It is deduced that the dynamic yield stress *τ*_0,*B*_ from rheometric investigations is not an appropriate parameter for the characterization of the effect of pre-shear time on rheological characteristics.

Figure 9b shows the evolution of mini-slump flow of the tested cement paste series. After mixing, i.e., 4.5 min after water addition, all pastes have a similar mini-slump flow measure of 250 ± 5 mm. The effect of pre-shear time on slump flow varies depending on solid volume fraction and superplasticizer addition. Again, in particular series 0.45_xSP without superplasticizer shows a significant increase in mini-slump flow with pre-shear time, which is assumed to be caused by a distinct breakage of agglomerates. Series 0.45, 0.48 and 0.52 show a less pronounced increase in the mini-slump flow value with pre-shear time. This is due to dispersing forces of superplasticizer and thus less attractive interparticle forces. Therefore, a pre-shear time of 30 s leads to a pronounced breakage of agglomerates which is only slightly increased with increased pre-shear time. Comparing the series 0.45, 0.48 and 0.52, an increase in the mini-slump flow values with an increase in solid volume fraction is observed: The paste 0.52 possesses the highest mini-slump flow values after pre-shear, which is explainable due to retardation and fluidization effects, caused by a higher amount of PCE.

### 4.3. Correlation of FBRM and Rheological Measurements

**Figure 10:**
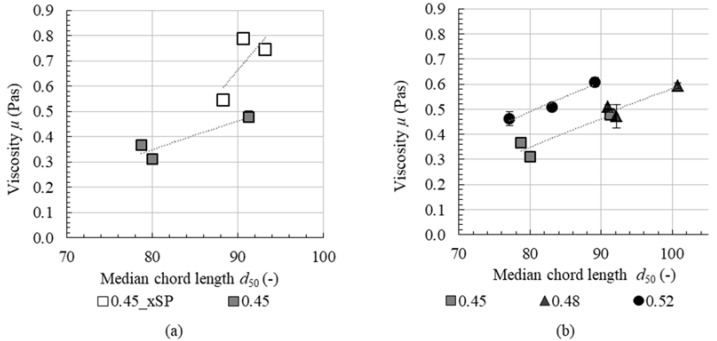
Correlation of the median chord length *d*_50_ and viscosity for pastes with and without superplasticizer (**a**) and different solid volume fractions (**b**).

In Section 4.1, the effect of various pre-shear times on the state of agglomeration in cement pastes was investigated. In Section 4.2 the rheological properties of cement pastes were evaluated after the application of various pre-shear times. In order to understand the fundamental mechanisms of fresh cement paste properties, a well-described relationship between microstructure and the rheological properties is required. Hence, the measurements from Section 4.1 and Section 4.2, namely median chord length, characterizing the agglomerate size, and apparent viscosity are correlated, Figure 10.

In Figure 10a cement paste with (0.45) and without (0.45_xSP) superplasticizer are shown. The median chord length for cement paste without superplasticizer (open squares) is slightly larger than with superplasticizer (grey squares), expressed in a shift of the data points to the right in the diagram. Concurrently, without superplasticizer the range of prevalent median chord lengths is smaller than for cement paste with superplasticizer. For both cement pastes, an agglomerated state, i.e., a higher chord length, is associated with an increase in viscosity. However, an increase in median chord length for cement paste without superplasticizer leads to an over-proportional increase in viscosity (higher slope for the indicated line of correlation), if compared to cement paste with superplasticizer. As already mentioned in Section 4.1, this is deduced from a stronger particle network when superplasticizer is absent. 

In Figure 10b, cement pastes with superplasticizer with various solid volume fractions are shown. Looking at each solid volume fraction separately, an increase in median chord length is accompanied by an increase in viscosity. It is assumed that the correlation of viscosity and the median chord length d_50_ has two main reasons:

- (a) Rheologically active water decreases with an increase in agglomerate size (water is trapped within the intra-agglomerate porosity). Therefore, with a higher median chord length the viscosity increases.
- (b) Larger agglomerates are accompanied by higher internal frictional forces during shear and thus higher apparent viscosity.

However, there is no general correlation between median chord length and viscosity independent of solid volume fraction. It is assumed that, depending on solid volume fraction and superplasticizer dosage, counteracting effects of packing density and dispersing on agglomeration kinetics are relevant. In order to distinguish these effects, further experiments are required. Nevertheless, with the combination of in-situ FBRM technique and high-sensitive rheometry, it finally can be stressed that increasing pre-shear time leads to a decrease in the number of contact points between cement particles, whereas these contact points themselves are dependent on solid volume fraction. Superplasticizer leads to weaker interparticle attraction between the cement particles and fast desagglomeration kinetics.

## 5. Conclusions

This contribution focuses on the effect of pre-shear on the state of agglomeration and rheological properties of cement paste. In total, four cement pastes were investigated. Three cement pastes with variations in solid volume fraction (0.45, 0.48, 0.52) contained superplasticizer. Additionally, one cement paste with a solid volume fraction of 0.45 without superplasticizer is evaluated. In experimental investigations, pre-shear times of 30 s, 90 s and 300 s were applied.

The state of flocculation is continuously measured in non-diluted samples using the focused beam reflectance measurement (FBRM) technique. An increase in time at rest after initial mixing leads to an increase in agglomerate size. Structure formation was attributed to the flocculation of particles and the formation of first hydration products. When subjected to shear, the progressive destruction of microstructure was assessed, expressed in a decrease in agglomerate size. The destruction of agglomerates under shear was more distinct, when superplasticizer was contained in the cement paste. It is assumed that superplasticizer reduces attractive interparticle forces and thus forming weaker agglomerates, which are broken more easily under shear. Moreover, the decrease in agglomerate size during shear was found to be depending on solid volume fraction, whereby low solid volume fractions were accompanied by a high decrease in agglomerate size. This effect was particularly pronounced for short periods of pre-shear. This can be attributed to a high number of particle contacts for high solid volume fractions, which make the destruction of agglomerates more difficult. For long pre-shear times, no severe difference in agglomerate decrease was observed for various solid volume fractions.

The rheological properties after pre-shear were measured using a plate-plate-rheometer. When increasing pre-shear time, a decrease in shear stress and viscosity was observed. This is deduced to a progressive destruction of the microstructure. Moreover, an increase in solid volume fraction and the absence of superplasticizer was accompanied by an increase in viscosity. Regardless of the solid volume fraction or amount of superplasticizer, no steady viscosity could be achieved when pre-shearing time was increased. Therefore, it was assumed that applied shear was not sufficient in order to destroy all agglomerates in the paste. Furthermore, based on the results from rheometer and mini-slump flow, it is concluded that mini-slump flow is better suited than rheometer with the applied shear-profile (Figure 3) to represent the state of agglomeration in cement paste since the material is exposed to lower shear during the test.

In order to understand the mechanisms of fresh cement paste properties, the viscosity was correlated with median chord length after shear. For all cement pastes, an increase in median chord length is accompanied with an increase in viscosity. For cement paste without superplasticizer, the increase in viscosity with increasing median chord length is even more distinct than for cement paste with superplasticizer, which is assumed to be attributable to stronger agglomerates.

Generally, the results of in-situ agglomeration measurements provide an understanding of the effect of pre-shear on agglomeration and on structural build-up of cementitious suspensions. This enables to optimize mixture compositions, e.g., by superplasticizer type and dosage in order to minimize agglomeration of particles. Since rheological properties are strongly dependent on the agglomerate network within the suspension, a change of shear history of agglomerated suspensions, i.e., mixing time or energy, results in altered rheological properties, which are therefore not directly comparable anymore. Hence, to ensure reproducibility and comparability, the mixing history should be chosen carefully. Moreover, in accordance with scientific publications and reports, the mixing history needs to be described in detail for the sake of comparable rheometric results.

## Figures and Tables

**Table 1:** Cement paste mixtures.

Mixture	w/c Ratio [−]	Solid Volume Fraction [−]	Cement [kg/m^3^]	Water [kg/m^3^] *	PCE [wt. % by Cement]
0.45_xSP	0.40	0.45	1399.5	550	-
0.45	0.40	0.45	1399.5	550	0.18
0.48	0.35	0.48	1492.8	520	0.60
0.52	0.30	0.52	1617.2	480	0.93

* The water content of the PCE was subtracted from the water amount to be added.

**Table 2:** Results of aggregate kinetics and rheological parameters for all testing series.

**Pre-Shear** **Time** **(s)**	**0.45_xSP**				**0.45**			
	$d_{50}$ **(μm)**	SF **(mm)**	$\tau_{0,B}$ **(Pa)**	$\mu_{P}$ **(Pa.s)**	$d_{50}$ **(μm)**	SF **(mm)**	$\tau_{0,B}$ **(Pa)**	$\mu_{P}$ **(Pa.s)**
30	90.65	151.0	62.5	0.79	91.25	239.0	13.3	0.48
90	93.21	158.0	58.8	0.75	78.71	251.0	13.2	0.37
300	88.28	182.5	48.4	0.48	80.04	261.5	11.9	0.27
**Pre-Shear** **Time** **(s)**	**0.48**				**0.52**			
	$d_{50}$ **(μm)**	SF **(mm)**	$\tau_{0,B}$ **(Pa)**	$\mu_{P}$ **(Pa.s)**	$d_{50}$ **(μm)**	SF **(mm)**	$\tau_{0,B}$ **(Pa)**	$\mu_{P}$ **(Pa.s)**
30	100.75	261.0	10.47	0.59	89.10	275.0	4.05	0.61
90	90.98	257.05	11.56	0.51	83.09	280.0	4.40	0.51
300	92.15	261.0	13.65	0.42	77.10	293.0	3.86	0.44
$d_{50}$: after shear; SF: slump flow; $\tau_{0,B}$: yield stress; $\mu_{P}$: plastic viscosity								
